# Reaction Produced by Cocain in Pressure Anesthesia

**Published:** 1905-02-15

**Authors:** E. T. Loeffler


### REACTION PRODUCED BY COCAIN IN PRESSURE ANESTHESIA.

BY E. T. LOEFFLER, B.S., D.D.S.

Read before the Detroit Dental Society.

Among the many substances that have been used as local anesthetics may be mentioned hydrochlorate, bromide and sulphate of ammonia; the carbonate and nitrate are without effect. Also copper sulphate and some of the iron salts, particularly the sesquioxide, produce anesthesia without coagulation at the point of application. Lead acetate is anesthetic, while the zinc salts are not. Among the organic salts or substances, hydroquinine, resorcine, antipyrine and the digitalis group are slightly active. Thallin, alcohol ether and glycerine have no action. The essential oils and similar substances have a remarkable action.

Most of the above mentioned drugs produce what is known as "painful anesthesia." Some substances, however, as cocain and beta eucain do not cause painful anesthesia and are followed by a contraction of the blood vessels. Whereas, those having a painful action cause a dilatation. They also have a caustic action, particularly hydroquinine. The same effect is true also of distilled water.

We can readily see that the objections to most of these agents are that, after the operation has been performed without pain there is a reaction, if too freely used, by return of the blood with great force, pressing upon the nerve endings; or after the use of caustic substances there may be partial death, so that in most cases the pain following is intense and prolonged. There may be loss of parts and severe hemorrhage. At times these agents may be employed with success, when no other means are at hand.

All these methods can be made much more efficient, if the surface be rendered bloodless by means of astringents or pressure.

I may be criticised for taking up very much time in explaining the action of cocain. But there is scarcely another drug that we have to deal with where there exists to much diversity of opinion in regard to its physiological action as in the case of this one. At any rate, a brief review of some of the most important views held on this subject may not be out of place at this time.

Just in what manner the contents of the tubuli are affected is not clear. Huber and others have demonstrated that the nerve fibrils pass between the odontoblastic cells and probably end there, at any rate, their extension into the tubuli of the dentin has not been proven.

There has been a good deal of discussion as to whether cocain acts equally on motor and sensory fibres, or whether it has a selective action.

Cushney states in his text book on pharmacology that cocain must be considered a general poison of protoplasm with some selective power for certain nerve tissue, but no specific affinity for sensory rather than motor terminations. He further states that it produces, besides a loss of sensation, a feeling of constriction and a distinct pallor and contraction of the vessels, the astringent feeling being easily explained by the fact that cocain is a poison of protoplasm, and the local contraction of the vessels may probably be due to the same cause.

The anesthesia produced by cocain is comparatively short, but varies with the strength of the solution applied and the vascularity of the part. In most cases the cocain is soon absorbed, and as this occurs the local action disappears and sensation returns.

The effects of cocain as a local anesthetic are to be ascribed to its destructive action on the protoplasm of the end organs. In some cases it may destroy their vitality. Muscles, nerves and nerve ends cease to contract or conduct stimuli when exposed for a short time to weak solutions of cocain. Ciliated epithelium cells, leucocytes, etc., become motionless. In some cases the final depression is preceded by a stage of increased movement. The paralysis of the sensory terminations is, therefore, to be regarded as only a particular instance of an almost general action upon living matter, induced by contact with cocain in sufficient amount.

Perhaps most of you are aware that cocain acts as a general anesthetic when very large doses are taken. Buxton, a noted English authority, states that its true action is analgesic, due, not to the vaso-motor constriction which it establishes, but to its influence upon the sensory nerve endings. If an area is rendered anemic and analgesic by cocain, the subsequent injection of pilocarpine will abrogate the anemia while the analgesia remains unaffected. Airlong has shown the same thing by dividing the superior cervical sympathetic of a rabbit on one side, the animal having been previously cocainized. Hypervascularity could thus be seen to exist simultaneously with analgesia.

In the use of cocain for obtunding sensitive dentin, or for the removal of the dental pulp, almost the first question that comes to our mind is: "What will be the reactions" This question received more or less attention when electricity first began to be used to increase the penetration of cocain into dentin. And now, since the introduction of mechanical means for forcing local anesthetics into tissues of the teeth under great pressure, the same question seems to be the all-important topic.

At the last meeting of the Michigan State Dental Association, held at Lansing, June last, when the members of the profession were being entertained by a number of practical demonstrations in the use of Myers' Obtunder, almost the first question that came to my mind was, "What will be the after-effects—will there be any complications?"

That action and reaction are equal and in opposite directions, is a phenomenon in physics, so common in our every-day experience, that its practical application in dentistry seems not only probable but possible.

After every surgical operation or injury there is a certain amount of reaction which is, as a rule, directly proportional to the extent of the operation or injury. This reaction we call "Hyperemia," and in case we have infection, inflammation.

Inasmuch as this question involves a knowledge of the action of the vaso-motor mechanism it might be well, at this time, to explain some differences that exist between vaso-constrictor and vaso-dilator nerves, which, in many respects, are particulary interesting for the reason that fibers of both varieties are found in one and the same anatomical nerve. The sciatic nerve is a good example of this class. That even a superficial knowledge of this fact is extremely important we can readily see, because the effects produced by ordinary methods of stimulation of the one might be wholly or in part masked by the effects produced by the stimulation of the other.

From the researches of Anrep and Cybulski, it has been found that the vaso-constrictors are more easily excited than the vaso-dilators. As a matter of illustration, they have clearly demonstrated that the simultaneous stimulation of the dilator and constrictor nerves, going to the sub-maxillary gland causes vaso-constriction, dilation appearing after stimulation ceases, for the after-effect of excitation is of shorter duration with the constrictors than with the dilators.

Again, Lepine and others have shown that warming increases and cooling diminishes the excitability of the constrictor fibers to a greater degree than in the case of the dilators. Also that the vaso-constrictors are more sensitive to rapidly repeated induction shocks of the same strength and less sensitive to single induction shocks than the vaso-dilators.

Furthermore, vaso-constrictors degenerate more rapidly than vaso-dilators, and the maximum effect is more quickly reached.

According to competent authority the the motor apparatus consists of three distinct classes of nerve cells. Cell bodies of the first class lie in the sympathetic ganglia, their neuraxons passing directly to the vessel walls; the second are situated at different levels of the cerebro-spinal axis, their neuraxons connecting with the ganglia by way of the spinal and cranial nerves; while the third are wholly within the bulb.

Perhaps this part of the discussion seems like too much of a deviation from the subject matter of this paper. But in order to comprehend the nature of the reactions which take place after operations upon living tissue, a superficial knowledge, at least, of the physiology of the vaso-motor mechanism can hardly be dispensed with.

There is, however, a lack of uniformity in the opinions held upon this all-important subject. From the experiments of Airlong, we are lead to believe that the astringent action of cocain is of reflex origin.

According to the statements of the other equally-important authorities, we are forced to the conclusion that concain does produce some temporary stimulation in the vaso-motor nerve endings which would, in a measure at least, account for some of the phenomena that take place after local anesthesia.

The latest researches on the action of cocain on the organs of special sense of taste, sight and smell warrant making the assertion that this drug has some selective action on sensory nerve terminations, particularly those of pain and touch. Other drugs as atropine, pilocarpine and muscarine have a similar selective action on nerve terminations.

The subject of pressure anesthesia is still in its infancy. More scientific as well as practical investigation is necessary to clear up some of these points and establish its general use. Our clinical experience with pressure anesthesia has been too brief and limited in a way to warrant making any definite statements, or to throw any great amount of light upon this subject. While in the majority of cases the operation seems to be a success, there are still too many failures proportionately to make its application universal.

The most important complications are hemorrhage, pain and possibly loss of pulp after obtunding dentin. Other objectionable features are anemic patients, or those possessing an idiosyncrasy against the use of cocain, or a hemorrhagic diathesis, to say nothing of cavities or teeth from which the saliva cannot be properly excluded.

I have instructed my students to keep an accurate record of all cases in which pressure anesthesia has been used, either for obtunding sensitive dentin or for the purpose of removing the pulp, and to have patients report at regular intervals during the year any complications that might arise.

Thus far the only unpleasant features we have had to contend with were excessive hemorrhage and very often secondary hemorrhage after removal of the pulp. If it is true that cocain produces its effect largely on account of its being a protoplasm poison, it would be a wise plan to limit the dosage and its action as much as possible. The more profound the paralysis the greater will be the reaction, and the more serious will be the danger from secondary hemorrhage and of hyperemia in case the pulp is not removed. The reason for this seems to be very clear, because the more paralysis we have in the blood vessels, the longer time will it take for them to regain their normal tonicity.

I should not fill any root canals at the same sitting after extirpation of the pulp, especially under pressure anesthesia. Thoroughly cleanse the root canals with dioxygen or other disinfectant, and leave a dressing of dry absorbent cotton for at least twenty-four hours to take care of any secondary hemorrhage or an accumulation of serum.

There is another source from which trouble may come, and that is infection. To guard against this contingency, extreme care ought to be exercised to see that the field of operation, solutions used, as well as instruments are thoroughly disinfected or if need be, sterilized before each operation. On account of the extreme pressure used, some of the cocain solution is apt to be forced into the apical region if there be any septic matter at the point of application, or in the tooth tissues, it will certainly be carried with the anesthetic into the apical space and there set up an active inflammation.

It might be well to call to your mind that we are dealing with tissues whose environment is such as to allow scarcely any room for expansion. So in case of hyperemia or active inflammation the symptons are always marked and the prognosis in many cases unfavorable. The old saying that "an ounce of prevention is worth a pound of cure," applies here with more force than in any other part of our work.

But how can we guard against some of these troublesome complications? That is a problem that can only be worked out by the united effort of every conscientious and ambitious worker in the profession.

The following are some of the more important suggestions, which, if rigidly adhered to, would tend to limit very materially the number of failures after pressure anesthesia.

1. A strict observance of all principles governing aseptic operations, and by this I mean that in every case the rubber dam ought to be applied and the cavity or tooth thoroughly dried and disinfected by the best possible means. All instruments should be sterilized and a fresh, sterile solution of cocain used in each case.

2. No more of the anesthetic should be used than is necessary to make the operation painless. To repeat what I said in a previous part of the paper, the more profound the paralysis, the more prolonged will be the time for recovery of the cells thus affected. Micro-organisms, which have gained entrance either through the solution used or through the blood and lymph channels, may gain a foothold before the cells of the tissue have fully recovered.

My first clinical experience with this method happened to be an easily accessible cavity, and on this account, perhaps, the time limit was greatly exceeded. So much so, that there seemed to be absolutely no feeling in the cavity or tooth for at least thirty minutes after the cocain was applied. This is a point, it seems to me, that ought to be closely watched. The cavity or the pulp should be tested at short intervals to determine whether sensation is lost or not. In case of the pulp, that test can only be applied when there is an exposure. Thus far, our experience would suggest that the maximum time limit be fixed at one minute when there is no leakage.

3. Either a weak solution or a limited amount of a stronger one is another principle that it would be well to bear in mind. By properly controlling the pressure we can, to a certain extent at least, limit the amount of the local anesthetic used. Just as in the administration of general anesthetics, pleasing results can be obtained by its careful and limited use that cannot be obtained in any other way. Cocain readily undergoes chemical changes in its composition so that solutions for use should be made fresh as required. Its anesthetic properties are destroyed by boiling, views to the contrary notwithstanding.

Inasmuch as cocain produces its characteristic effects by paralyzing the nerve endings, the less constriction there is produced in the blood vessels the better. Because the reaction depends very largely upon the amount of disturbance there has been produced in the blood supply. For this reason I cannot see what particular advantage would be gained in the end by using a special astringent, such as adrenalin chloride. So powerful an astringent as this makes a bloodless operation a possibility, but it also greatly increases the dangers from secondary hemorrhage and not infrequently other serious complications. Two cases, at least, that I have in mind, both operations upon the nose and in which a combination of cocain and adrenalin chloride were used, have clearly demonstrated to my mind at any rate that there is more truth than fiction in the above statement. In the one case there was an unusual amount of trouble from secondary hemorrhage. In the other, erysipelas was the complication, brought about, undoubtedly, through the profound debility produced in the tissue cells, thus rendering the parts more susceptible to infection.

